# Sustainable nutrition systems with alternative proteins for low-impact food future

**DOI:** 10.3389/fnut.2026.1883773

**Published:** 2026-07-14

**Authors:** Sultan Matar Almutairi

**Affiliations:** Department of Basic Health Sciences, College of Applied Medical Sciences, Qassim University, Buraidah, Saudi Arabia

**Keywords:** alternative proteins, circular economy, consumer acceptance, environmental sustainability, food policy, insect-based protein, life cycle assessment, microbial protein

## Abstract

Feeding the world's increasing population is an enormous task for our current food system. Traditional animal agriculture harms the environment through high land and water use and significant greenhouse gas emission. Sustainable food systems will promote a low-impact food future through the use of alternative protein sources. Alternative protein sources include plant-based, microbially based, and insect-based proteins. Examples of those types of alternative proteins are: plant based includes peas, soy, and hemp; microbially based includes fungi and bacteria; and insect protein. The purpose of this article is to utilize life cycle assessment (LCA) data to assess the environmental sustainability of a range of alternative protein sources compared with conventional sources of protein. Another objective of the article is to discuss new technologies available for the processing and production of foods from alternative proteins, including fermentation and low-impact processing techniques, as well as biodegradable packaging materials and other sustainable packaging options that can mitigate waste generated by food packaging. The acceptance of these products by consumers will also be discussed in relation to the overall sustainability of alternative protein sources, as studies have found that many consumers would like to try new foods; however, taste, price, and familiarity are the three main reasons consumers typically do not purchase alternative protein products. There are many factors affecting a person's decisions to adopt a new way of eating, including risk of deficiencies in Vitamin B_12_, Iron, and Omega-3 fatty acids, along with considerations of food processing and socio-economic factors. Policies and regulations created by government agencies, in addition to providing financial support through subsidies; regulations that set clear labeling parameters; and economic considerations (i.e., price and market growth), which affect the purchasing decisions of consumers. The market for plant-based meat continues to grow quickly. As such, there is a need for future research into improving the nutritional content and flavor of these products, as well as increasing their production capabilities.This review uniquely integrates environmental life cycle assessment, nutritional adequacy,consumer behavior, and policy frameworks to compare plant-based, microbial, and insect-based proteins within a circular food systems perspective.

## 1 Introduction

The global food system is confronted with the challenge that has to be improved because food production produces negative impacts on the planet. Agriculture from animals contributes significantly to this environmental harm through producing greenhouse gases and making up approximately four times the total greenhouse emissions of the transport sector (1, 2). Additionally, animal farming and animal products require significant resources, including land and water use. For example, it requires around 20,000 L of water to produce 1 kg of beef protein, and it consumes approximately 150–250 square meters of land per year per beef animal. On the other hand, as the global population continues to increase, it is projected to reach almost 10 billion people by 2050, increasing food demand by 50%−70% from 2021 to 2050 (1, 3, 4). A viable solution for keeping food availability high without harming the planet is through sustainable nutrition systems, which focus on providing healthy food while creating, conserving, and protecting natural resources (5). Alternative proteins are one of the key elements in these systems.

The use of alternative proteins is not a new concept. Humans have consumed things like beans and tofu as sources of nutrition since the beginning of humanity. However, we now have access to a much larger variety of alternative proteins than ever before due to the work being done by scientists and companies to develop new types of protein as well as improve existing plant protein sources and low-impact food futures (6). Some examples of these proteins are those derived from microorganisms and insects, as well as plant-based products. The ultimate goal of these protein representative companies is to replace a portion of our consumption of meat that originates from animal sources. Currently, animal products make up approximately 60% of the protein consumed globally; however, they also occupy 80% of the total agricultural land resources worldwide (2, 7), thus offering an inefficient means to meet our dietary protein needs. Therefore, the notion is not to eliminate all forms of animal-based meat; rather, the desire is for people to reduce their overall consumption of meat (8). Additionally, if everyone in the United States replaced just one beef meal a week with a plant-based meal, it would provide the equivalent carbon savings of removing 10 million vehicles from the road (9). Blue foods that include aquatic species also offer another pathway to sustainable protein (10).

Nonetheless, there are flaws in alternative proteins, as some can be highly processed and include additives; other alternatives can also have nutritional concerns. One nutritional problem is that not all plant proteins are complete, meaning that they lack some of the nine essential amino acids (11). By combining several sources of protein, like rice and beans together, it is possible to create a complete protein made with various foods. Fortifying some of the new plant protein products sold is also common practice; A lot of the time, vitamin B12 (a vitamin too low in most plant foods) gets added to the product during the production process (12). Therefore, according to Lonnie and Johnstone (12), approximately 20%−30% of the world's population is at risk of having a Vitamin B1_2_ deficiency due to switching from an Omega-3 to an all Plant-Based or unfortified food diet, so Fortification will continue to be a critical Health Product in our future (13).

Another major challenge to consumer acceptance is how many people enjoy eating meat as well as the feel of meat products on their mouths; therefore, there are still existing foods that mimic animal flesh but do not yet have similar mouth-feel characteristics. Surveys have revealed that approximately 30% to 40% of European and North American populations have tried consuming vegetable-based food alternatives in place of traditional meat/products. However, only approximately 10% to 15% consumed vegetable meat alternatives on a consistent daily basis (14). Surveys indicate that one of the most common reasons given by consumers for not continuing to purchase these products is due to their lack of taste, both in terms of flavor and texture (15). However, technology continues to advance quickly. Technology is used to connect the production, animal nutrition, and human health under a “One Nutrition” framework (16). As many of these products use similar ingredients to current meat alternatives, companies are pouring immense amounts of money into research/development in order to make products more closely resemble traditional beef or chicken in terms of texture, flavor, cook properties. In 2024, total global investment in non-animal-source alternative protein reached approximately 3 billion U.S. dollars (17). Prices will decrease as production increases, but currently, many plant-based alternatives (i.e., Veggie Burgers) can be priced 20%−30% higher than their beef counterparts (18). The dietary guidelines are helping increasingly to incorporate sustainability indices like WISH (World Index for Sustainability and Health) to evaluate whole diets (19).

## 2 Source of alternative protein

### 2.1 Plant-based proteins

Alternative proteins have long been sourced from plants, the traditional means of protein consumption. The wasted food and crop biomass have been used to develop feeds for cattle by way of beans, lentils, and peas for many years. These types of food are inexpensive, nutritious, and beneficial to the environment. Plants can be produced using less land and water than livestock (3). They also create fewer greenhouse gas emissions overall than do animal products. Although some plant foods do have a higher environmental impact than others, for example, almonds due to their high water usage, most plant foods are better for the environment compared to meat. Some people have concerns about how soy is produced, particularly because a large proportion of the soy being produced comes at the expense of cutting down trees in the Amazon, which is primarily used as feed for animals. However, when soy is consumed directly by human beings instead of being fed to animals, it is much more efficient (20). Another excellent option for plant protein is peas; peas are often utilized in the production of plant-based alternative milks and burgers. A case study from France identified the barriers and levers for plant protein development (21). Plant-based diets are a continuum rather than a single dietary pattern. The term “plant-based” does not necessarily mean “vegan”, a common misconception, but includes flexitarian, vegetarian, and pescatarian patterns. Understanding this spectrum matters because it allows tailoring of nutritional advice. Vegans require B1_2_ and iron supplementation, while flexitarians may only need modest dietary adjustments.

Hemp is considered a newer plant source for protein. Basic information on hemp is as follows: Hemp seeds are highly nutritious, as they are full of protein, beneficial fats, fiber, etc. Hemp is easy to grow with little resource consumption (22).

Additionally, hemp helps improve soil health. Researchers are creating meat substitutes out of hemp (23). Therefore, the future for hemp meat substitutes looks good! There are many other plant sources for protein; examples include fava beans, chickpeas, and lupins, but some companies are using leaves to extract protein. The extraction of protein from leaves is a new area of research, but researchers are developing ways to extract protein from spinach and alfalfa leaves (24). Plant proteins are no longer only for vegetarians; more meat eaters are purchasing them. Plant proteins do not contain any cholesterol, have low amounts of saturated fat, and provide fiber and antioxidants to support heart and intestinal health (25). However, the downside of using plant protein is that there is often an earthy or “bean-like” flavor to it, which may not appeal to everyone. These plant proteins can have their flavor removed through processing; however, the processing adds cost and excess usage of energy.

Texture is another challenge in replicating a meat product, as meat contains a unique fibrous structure. The challenge for food scientists is to create this fibrous structure from plant material, and they continue to make advances in the field. One technique that is being used to make plant-based proteins look and feel like chicken or beef is extrusion processing. There are very realistic products that exist; however, the level of realism will continue to evolve with advances in technology. Plant proteins can be used as ingredients in soups, stews, and stir-fries, or they can provide a meat alternative for tacos and pasta sauce. This versatility of plant protein is a key strength. Increasing the production of plant proteins is also beneficial to farmers; when crops are grown in rotation with legumes, soil productivity increases, and synthetic fertilizer application decreases. This also interrupts pest life cycles. Thus, plant proteins will positively impact all systems associated with growing food (26). Therefore, plant-based proteins represent an already well-defined, functional, healthy, sustainable, and widely accepted solution. Alternative protein sources like legumes also show promise for livestock feed (27). For example, ultra-processed plant-based meats can contain up to 500 to 800mg of sodium per serving (vs. 50 to 100 mg in unprocessed legumes), and some studies associate high ultra-processed plant food intake with a 12%−20% increase in all-cause mortality (28). The overall score reflects the relative contribution of plant vs. animal foods, with positive points for plant foods and negative points for animal foods.

### 2.2 Microbial proteins

Microbial proteins have been around for quite a while; they come from microbes, which are very small living things like fungi, bacteria, and algae. They grow so quickly that they can double their mass within just a couple of hours, resulting in being much more efficient than other types of protein sources. One major advantage of microbial protein sources is that they do not require as much land or water to cultivate. Instead, microbes can be grown under controlled conditions, such as in large tanks or bioreactors, sometimes using waste materials (29). Circular Bio-Economy Models are the key focus for the waste-derived feedstocks (30). Microbial proteins are also called single-cell proteins. The reason for this is that each organism or microbe is essentially one cell. Fungi are one of the most common sources of microbial protein. Microbial protein systems can achieve very low carbon footprints (31). A well-known example of a type of fungal protein is mycoprotein, which is produced from the fungus *Fusarium venenatum*, and is sold under the brand name Quorn. Mycoprotein has a texture comparable to meat; it is considered to be a high-quality protein source and also has a fair amount of dietary fiber, which can aid in digestion. In addition, producing mycoprotein takes far less land compared to producing beef or chicken, as well as producing fewer greenhouse gas emissions (32). Basidiomycetes, which are comprised of mushrooms and other types of fungi, can also produce a type of fungal protein. Researchers can produce mycelium (the root-like portion of a mushroom) to provide a meat-substituting product (33). The variety of fungi provides opportunities for the sustainable food system development (34).

**Figure 1:**
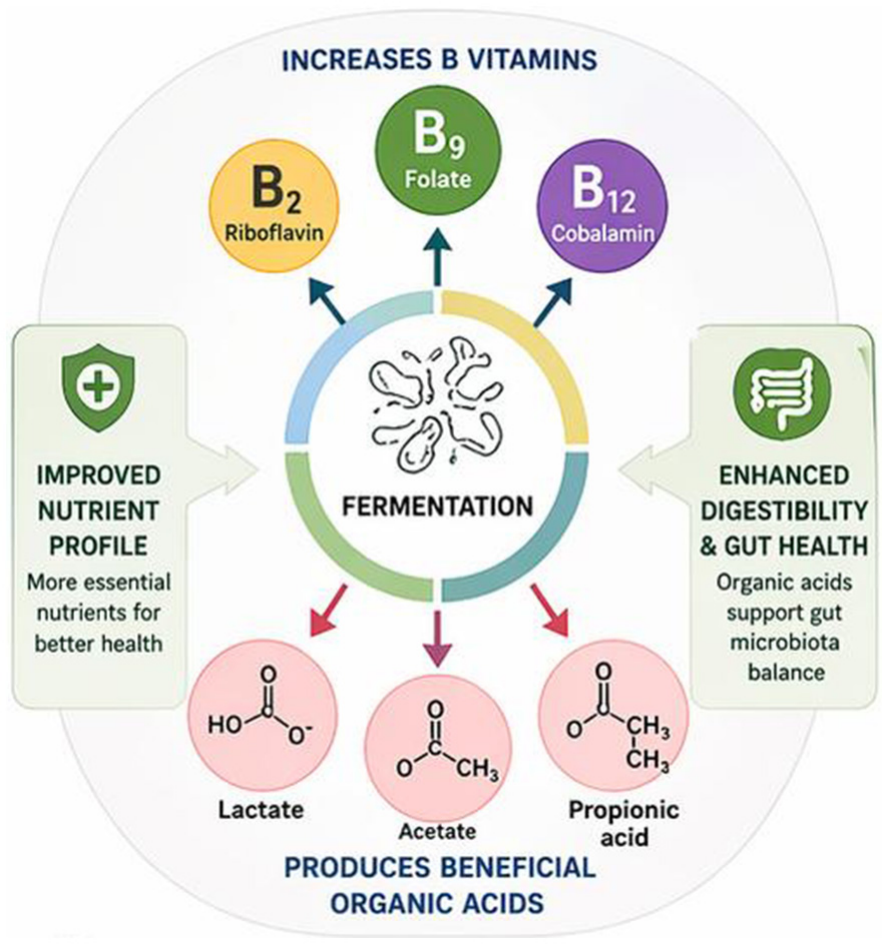
Fermentation naturally boosts nutrients and creates gut-friendly compounds.

Bacteria are yet another source of protein. Compared to fungi, they reproduce more rapidly. Certain types of bacteria can use hydrogen gas and carbon dioxide to create protein. This method of producing protein is known as “power-to-food” and does not require sunlight or sugar, so it can be used in virtually any location (including space or deserts). Bacterial proteins are very pure and contain all of the essential amino acids that humans need. Bacteria also do not produce any of the plant-based compounds that lead to allergic reactions (35). However, bacterial protein is also very expensive to produce because the technology used to create it is still relatively new. Like bacteria, algae are classified as microorganisms. This category includes microalgae, such as spirulina and chlorella, which humans have consumed for centuries due to their high nutritional value (spirulina is composed of 60%−70% protein) and health-and-heart-sustaining properties from Omega-3 fatty acids. Algae can be cultivated with very few resources (sunlight, water, and a limited number of nutrients) and can be grown in either open ponds or closed tanks. In addition, as algae continue to grow, they absorb CO_2_ from the atmosphere, which is beneficial for the global climate (36). Unfortunately, some people may find that they do not enjoy algae due to its typically strong taste, which can be described as either earthy or fishy. This will limit how much algae can be utilized in certain products, e.g., smoothies or supplements. Algae and fermentation-derived feeds are also explored for livestock feed (37) (Figure 1).

The diagram illustrates the benefits of fermentation on the nutritional value of alternative proteins. Fermentation offers key vitamins that include riboflavin (Vitamin B_2_), folate (Vitamin B9), and cobalamin (Vitamin B1_2_). Fermentation also produces key organic acids such as lactate, acetate, and propionic acid. In addition, the enzyme phytase breaks down antinutrients (e.g., phytic acid) that limit mineral absorption for minerals like iron, zinc, and calcium (38, 39), and Gabriele et al. (40) include this information in their research.

Microbial proteins have many benefits. One major benefit is that they have a much lower impact on the environment. Microbial proteins require very little land to grow, and they can grow vertically in tanks, which saves space. Life cycle assessments show that microbial proteins are better for the environment than livestock, with one study showing that microbial protein has 90% lower carbon emissions than beef (41). Another study found that fossil fuels are used as energy sources in the production of microbial proteins (42). Microbial proteins have many benefits. One major benefit is that they have a much lower impact on the environment. Microbial proteins require very little land to grow, and they can grow vertically in tanks, which saves space. Life cycle assessments show that microbial proteins are better for the environment than livestock, with one study showing that microbial protein has 90% lower carbon emissions than beef (41). Another study found that fossil fuels are used as energy sources in the production of microbial proteins (42). These indices help distinguish diets rich in whole plant foods from those dominated by refined plant products, which may explain inconsistencies in health outcomes across studies. For example, higher plant-based diet index PDI scores are consistently linked to a 20%−30% lower risk of type 2 diabetes.

### 2.3 Insect-based protein

In many different cultures, including those in Africa, Asia, and South America, insects are consumed as a source of food. Most notably, crickets, mealworms, and black soldier fly larvae are being researched and used as a food source. Insects require little water, space, and feed compared to other protein sources and have a rapid growth cycle. Additionally, insects can use food waste (such as vegetable scraps or grains) to convert them into protein that is both safe and nutritious to eat (43). This practice is good for our environment. Crickets are among the most commonly consumed insect foods; their composition is approximately 60%−70% protein based on their dry weight. Mealworms are eaten both whole (often roasted) or as flour. When eaten whole, mealworms are frequently eaten as snacks because they have an earthy taste. Black soldier fly larvae have a very high rate of efficiency when consuming waste; they are capable of consuming just about any organic waste, with the exception of non-organic, industrial-grade recycling plant waste, including human waste and food waste. In addition to being an excellent source of protein, black soldier fly larvae also contain a significant amount of fat. At present, most black soldier fly larvae products are used for animal feed, but research is being conducted on how black soldier fly larvae products could also be consumed by humans (44). Alternatively, insect-based feeds are also used for chicken farming (45).

The small environmental impact of insect farming. The life cycle assessment of mealworms showed a low impact on the environment when comparing them to pigs and chickens. Mealworms require less land and water than either of those two animals and produce significantly less greenhouse gas emissions than either of those two (46). A study on cricket farming also found very similar results about crickets needing six times less feed than cattle, producing 100 times less greenhouse gas per kilogram of protein (47), and reproducing rapidly by laying hundreds of eggs at one time, allowing for faster scaling of production. Socially, there are many positive aspects of insect farming. It can be conducted in small spaces and does not require expensive equipment, which allows poor communities to participate. Insects can be grown in homes or backyards and provide both food and income. In Singapore, there is a decentralized system that uses black soldier flies to consume discarded food (processing food waste) and produce food (animal protein) and fertilizer (plant). This reduces landfills and generates value (48). Many cities can duplicate this type of system.

There exist multiple barriers to edible insect consumption. First, consumer disgust is likely the largest barrier inhibiting the purchasing of edible insects. Second, many insects are very strongly flavored. Processed insect foods, however, can be used to disguise these strong flavors through processing (for example, using ground insect flour as an ingredient in foods that do not contain visible insect products) (49). Additionally, currently, many insect foods are available at a high price due to limited supply. However, as more consumers exhibit interest in edible insects, food manufacturers will steadily increase their production of edible insects, and food prices will be lower. Food safety is critical, as insects may harbor pathogenic microorganisms (50). Insects used for food must be produced to strict hygiene standards, and all insects must be cooked or processed to kill any harmful microorganisms before they are sold to consumers. In India, the nutrition transition has displaced traditional legume and millet-based diets with refined grains and animal products, increasing cardiovascular disease risk. Similarly, in many African nations, globalization promotes processed foods over indigenous leafy vegetables and pulses.

## 3 Environmental sustainability assessment of protein sources

To create a fair comparison of protein sources, we will draw on life cycle assessment (LCA) data as our guide. An LCA analyses the impacts of a product over its entire lifecycle. This includes, but is not limited to, the farming, processing, transportation, and disposal phases of the product's lifecycle. During LCAs, many different types of environmental effects will be measured, some examples of which are: carbon footprint, water use, land use, and more. Key findings of recent LCA studies of animal protein (land-based) compared to plant, microbial, and insect-based proteins are summarized in Table 1. The data in Table 1 demonstrates the consistent trend that animal protein has a significantly greater impact than that of plant, microbial, or insect-based protein sources. However, not all studies show consistent reductions in inflammatory markers, and confounding factors such as BMI and overall lifestyle may influence results. This 10%−25% LDL reduction is clinically meaningful. By comparison, moderate-intensity statin therapy typically lowers LDL by 30%−50%. Thus, while plant-based diets are not a replacement for pharmacotherapy in high-risk individuals, they provide a valuable non-pharmacological strategy for primary prevention and adjunctive therapy.

**Table 1:** Comparative environmental impacts of protein sources (per kg protein).

Protein source	GHG emissions (kg CO_2_)	Land use (m^2^/year)	Water use (liters)	References
Beef	300–500	150–250	15,000–20,000	(1)
Pork	50–100	10–15	5,000–7,000	(100)
Chicken	30–50	5–10	3,000–5,000	(92)
Tofu (soy)	10–20	2–4	1,000–2,000	(3)
Pea protein	8–15	1–3	500–1,500	(77)
Mycoprotein	5–10	0.5–1	100–500	(32)
Spirulina	5–8	0.2–0.5	100–300	(42)
Mealworms	10–20	1–2	100–500	(46)
Crickets	8–15	0.5–1	50–200	(47)

**Figure 2:**
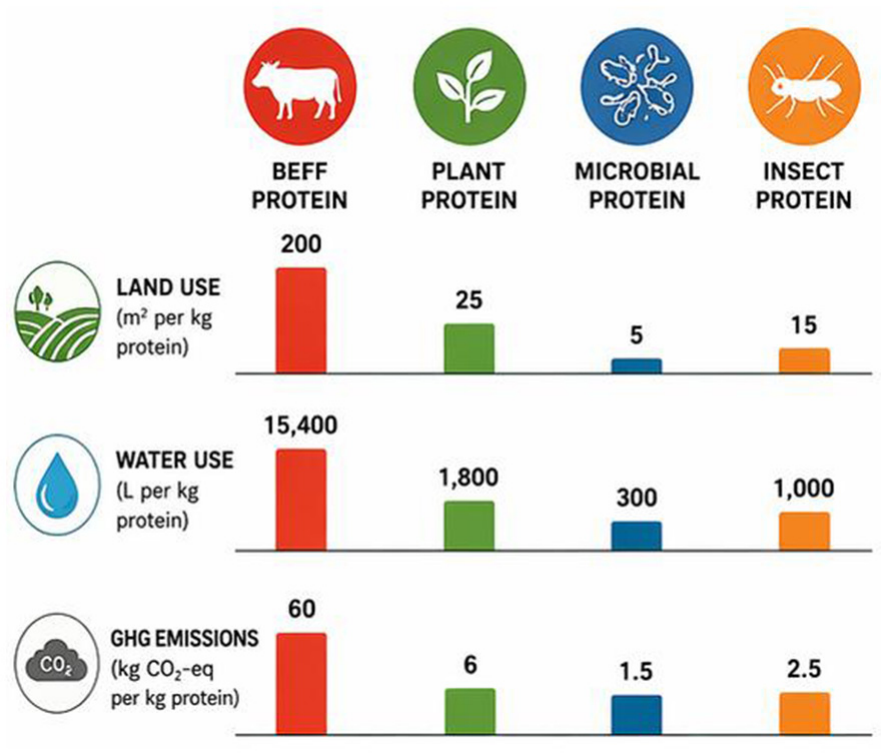
Comparing environmental sustainability of protein sources.

The use of certain plant proteins, such as soy and pea, produces less waste than animal proteins. However, insects are the least polluting alternative protein. Still, there is a huge range of impacts depending on how the proteins are produced, so it is important to look at the entire production system. For example, the amount of fertilizer used to grow peas can greatly increase GHG emissions. Additionally, the methods of processing soybeans into tofu and peas into protein powder require considerable energy and, therefore, must also be included in the total impact of each protein's lifecycle (51). While animal products consume large amounts of water (for drinking, hygienic purposes, and to grow feed), beef is the most water-intensive (for example, 20,000 L of water would be required to produce 1 kg of beef protein). Microbial proteins (in many cases) do not use much water (certain bacteria can be grown using gas without water). This offers considerable water savings (41). Land use is another significant impact created by livestock agriculture. Livestock agriculture uses approximately one-third of the world's land area; much of this land is either grazing land or cropland used to produce livestock feed. The area of land required to produce alternative proteins is far smaller than that required for livestock production. Therefore, the land formerly used for livestock agriculture can be rewilded. Rewilding forests and grasslands will assist with carbon sequestration and biodiversity enhancement (2). The importance of the food disadoption for environmental sustainability is overlooked and critical for new foods (52). Wasted food and crop biomass can be used to develop novel feeds for cattle (50). While colorectal cancer shows the strongest and most consistent protective association, evidence for breast and prostate cancers is more mixed. Some studies report null or weak inverse associations, possibly due to heterogeneity in dietary patterns and study designs (Figure 2).

This decision flowchart evaluates protein supply sources using four key environmental indicators: greenhouse gas emissions, land use, water use, and energy use. The decision flowchart guides the evaluation from raw material (plant, microbe, insect, or animal), through processing methods, to final rating for environmental impact (low, medium, or high). Blue foods, including low-trophic aquaculture, offer additional opportunities for sustainable proteins (53). However, some studies also report reductions in Bacteroides abundance, which may have implications for protein fermentation and host metabolism. A balanced view acknowledges that while butyrate-producing species generally increase, other taxa may decline.

While alternative protein sources are better for the environment than animal sources, we must be cautious. For example, almond milk has become widely accepted as an alternative to dairy products, but almond trees require a significant amount of water to produce almonds. In addition, most plant-based meats contain oils (such as palm or coconut) associated with high rates of deforestation (54). For instance, a pea-based burger may have little to no impact on sustainability; however, if it contains palm oil or coconut oil that has been imported, then it would not be as sustainable (55). Energy, water, and food security are valuable for specific crops (56). Another critical aspect to consider is how nutritious each type of protein is. While comparing protein sources based on the number of grams of protein in 1 kg will give you an overall idea of protein quality, it will not provide a complete picture. Some proteins are more bioavailable (digestible) than others. Animal protein generally has a 90%−95% bioavailability, whereas plant-based protein typically only has a 70%−85% bioavailability but can be enhanced with various cooking methods (e.g., boiling vs. cooking in the microwave). For example, roasting or grinding grains before culinary preparations increases their digestibility. Fermented foods also increase bioavailability. Microbial protein has been found to have very high bioavailability (typically >90%); therefore, 1 g of microbial protein will provide greater nutritional benefit than 1 g of plant-based protein (57).

Scientists have created improved ways of measuring protein sustainability using a “farm-to-faeces” approach. This method measures the amount of protein consumed from the field through the digestion and metabolism of the proteins. In other words, the impact of each gram of usable protein would be larger (51). Other researchers used a multi-criteria decision analysis in some of their studies to evaluate the various impacts on a protein's sustainability by combining carbon, water, land, and nutritional metrics. The researchers used this method to create more equitable measures of protein sustainability (58). Multi-criteria approaches are especially useful for assessing trade-offs in complex food systems. Additional consideration in the evaluation of protein sustainability is energy use from cooking and processing the proteins. A researcher conducted a study in Sweden to investigate (59). In the results, the researcher observed that most plant-based meals used less energy to cook. Still, some meat substitutes required a lot of processing and thus had a larger energy footprint than either a plant-based or an unprocessed animal protein would have. However, if cooking and processing of meat substitutes are done with renewable energy sources, then both would be equally sustainable (60).

There is a need to adopt new foods and discontinue previous ones in order to help the environment. If one starts adding to their diet but does not give up, they are adding to the overall detrimental impact of the food system (52). Education and policy should focus on encouraging the discontinuation of previous harmful dietary practices in favor of sustainable alternatives. Another consideration is rebound effects, which are when an individual perceives that they are doing something “good” and, therefore, they eat or spend more. This can negate the aforementioned benefits associated with this behavior (61). Despite these considerations, research demonstrates that there are significantly lower environmental impacts associated with the consumption of alternative proteins (most are significantly lower than animal-derived proteins). On average, plant-based proteins use 50% to 90% less land, water, and produce 50% to 90% fewer GHG. Microbial and insect proteins also have even lower environmental impacts; however, they are not as widely developed. A flexible approach that encourages the widespread use of varied alternative proteins will contribute to creating a more resilient food system (9). A variety of protein sources supports nutritional security across various population groups (62). The Mediterranean diet offers a model of sustainability along with cultural relevance (63). There is currently a very small amount of edible insect food on the market for consumers; however, this could change quickly (7). Many regulatory frameworks are evolving in many jurisdictions for insect-based foods (64). It is important to acknowledge that microbiome responses to plant-based diets vary considerably across individuals due to differences in baseline microbiota composition, genetics, and geography.

## 4 Development of alternative protein-based food products

It's hard to make tasty alternatives to traditional foods using alternative protein sources. When creating alternative protein products, you want the final product to be both nutritious and have a nice texture or mouth feel (65). Food scientists have been doing a lot of research on this topic and have developed a number of different techniques/methods to accomplish this. The following paragraphs will discuss the different areas of food science used to develop alternative protein products. They will include examples of different types of alternative protein products and their sources in Table 2.

**Table 2:** Alternative protein products and their sources.

Product category	Example product	Primary protein source	Processing method	References
Meat analog	Plant-based burger	Pea or soy protein	Extrusion	(25, 100)
Meat analog	Mycoprotein nugget	Fungal mycelium	Fermentation + texturizing	(32)
Dairy analog	Almond milk	Almonds	Wet-miling + enzyme treatment	(101)
Dairy analog	Coconut yogurt	Coconut	Fermentation	(38)
Egg analog	Aquafaba meringue	Chickpea water	Whipping	(68)
Seafood analog	Plant-based tuna	Soy and wheat protein	Extrusion + flavoring	(102)
Snack	Cricket protein bar	Cricket flour	Grinding + mixing	(43, 47)
Beverage	Fermented pea drink	Pea protein	Fermentation	(39)

Fermentation is an important part of food production; it has been a method of food preparation for thousands of years, as fermentation can alter the flavor and texture (in addition to removing off-flavors from food). For instance, when pea protein is fermented, it becomes less “beany” in flavor. Fermentation also adds live beneficial bacteria, which are probiotics (good for gut health), whereas the different microbes that are used to ferment foods provide varying results. Some types of fermentation can create a meat-like or cheese-like flavor; other methods will create a creamy texture. Fermentation is being employed in the creation of tempeh, miso, and many other plant-based dairy products (39).

The flowchart depicts the conversion of raw materials into products via microbial action. The major processes for turning raw substrates into fermented products include inoculation, fermentation temperature/pH/timing by fermentation tank, separating and collecting finished fermented product from its respective fermentation, postharvest processing, and production of vitamins B_2_, B_9_ and B1_2_ and organic acids (lactate, acetate, propionic). Fermentation can enhance B1_2_ content in plant-based products (66).

**Figure 3:**
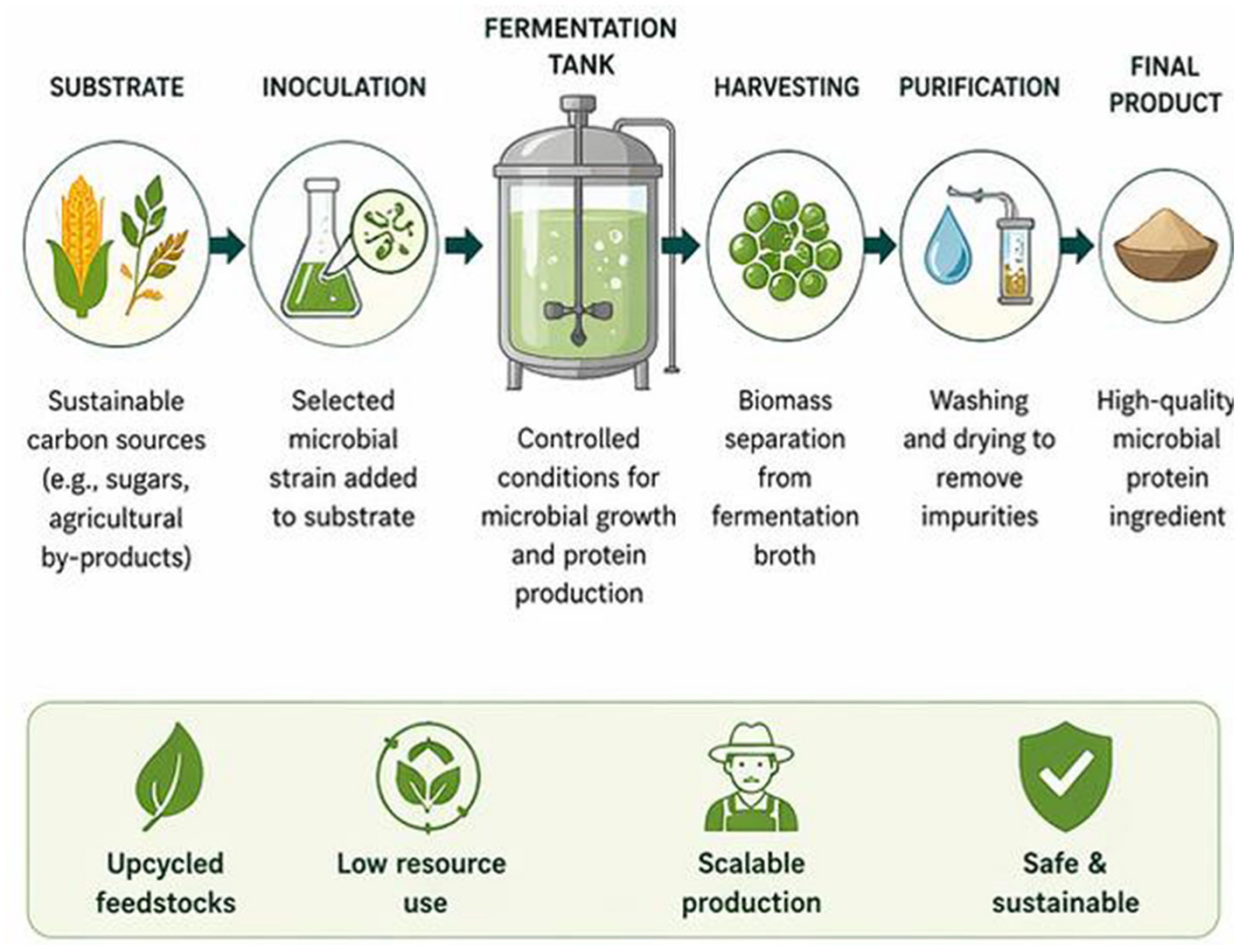
Fermentation process of microbial protein production.

Another new opportunity for innovation in fermentation is through the use of biomass fermentation, which involves the growth of large quantities of fungal mycelium; the mycelium itself serves as the food product. Quorn is an example of this method; the mycelium is harvested, mixed with a binder, and textured to form a meat alternative (32). Precision fermentation represents an even more recent advancement in fermentation technology by utilizing genetically-engineered microbes to produce specific proteins. As an example, yeast can be modified to produce casein, which is the primary protein found in milk. The role of fermentation in a sustainable future includes waste streams (40). The modified casein can then be mixed with plant-based fats and sugars to create commercial dairy products without cows. This type of product is referred to as animal-free dairy, and it is very similar in taste and meltability to conventional cheese, with many consumers favoring the animal-free cheese product over the traditional cheese product. However, it is important to note that precision fermentation can be very expensive, and it is still facing regulatory challenges within the food industry (17). The consumer preferences for clean labels are pushing companies to simplify ingredients (67) (Figure 3).

Flavor is the third principle in this model. Flavor is important regardless of its sustainability. Plant-based proteins can exhibit off-flavors due in part to saponins and phenols. They can be quite bitter or taste beany, and certain methods of processing will typically help to eliminate some of these compounds. One way of processing will include washing the protein with either water or alcohol to help remove some of the off-flavors. Another method would be to utilize enzyme activity to degrade the off-flavors from the protein. Another way to hide the off-flavor is to add other flavors that can mask it. Iron deficiency anemia rates are not significantly elevated in well-nourished populations with diverse diets consuming well-planned plant-based diets.

Additionally, many of the plant-based meats have very long ingredient lists, which are partially attributable to flavoring chemicals (28). Many of the current ingredients used for plant-based meat analog consist of new and unique ingredients. For example, Aquafaba, which is the liquid from cooking chickpeas (chickpea water), has been found to have similar physical properties to egg white when whipped. As such, Aquafaba is excellent for making meringues and mayonnaise, and can be considered a food source from what would otherwise be considered a waste product. For this reason, Aquafaba is highly regarded as a sustainable food (68). Another ingredient that contains high-quality protein is what is referred to as “leaf protein.” The leaves of many crops, such as sugar beets or alfalfa, are often discarded without being utilized in any form. However, recent developments in the extraction of leaf protein from foliage are now allowing for the recovery of high-quality protein from those discarded leaves. The resulting leaf protein powder has a unique green color and grassy taste, limiting some of its use to producing pesto and the addition of the leaf protein powder to smoothies (24).

The trend of low-impact processing is expanding, with improved alternatives that use ultrasound to destructively heat protein cell walls using sound waves, releasing proteins without heat, and pulsed electric fields to extract fat from oils through very short bursts of electricity (69). An alternative, the use of enzymes instead of chemicals, provides specific and clean extraction methods using low temperatures while decreasing waste and energy costs (70). Additionally, the foodservice industry is transforming as well, with many meal service providers such as schools, hospitals, and office cafeterias providing thousands of meals daily. Simple changes made to improve food sustainability can have a tremendous impact. For example, the use of a menu scoring system is being implemented by some service providers, allowing them to rate meals by their environmental impact and promote low-impact meals and reduce the frequency of higher-impact meals served. In Italy, for example, the introduction of a menu scoring system has been shown to reduce the carbon footprint of catered meals without complaints from the public (71). Similarly, in the United States, introducing the practice of reducing the amount of meat protein and increasing the amount of whole grains used in school lunches has resulted in much lower environmental impacts (72). Thus, the development of new products is occurring through finding better ways to use existing foods, as well.

## 5 Sustainable packaging for alternative protein products

Packaging is too often overlooked. Yet it is crucial to food products as it keeps them fresh and safe while providing information regarding the product. However, packaging contributes to the waste that pollutes our environment, as most food packages are made of plastic, which is derived from fossil fuels and takes a long time to disintegrate - often ending up in our oceans and affecting wildlife. Some biodegradable plastics require industrial composting facilities and do not breakdown in home compost. If this is not done, the plastic can remain for an extended period of time (73). Challenges remain, including regional differences in agricultural practices, cultural dietary preferences, and the economic feasibility of large-scale dietary shifts.

Recycled plastic is an alternative option because it decreases the demand for virgin plastic and helps eliminate waste from landfills. The number of companies increasing their recycled plastic content has risen greatly. Some of them utilize entirely recycled plastic. Despite the above-stated limitations, recycled plastic has less of an impact on the environment than new plastic (74). Paper and cardboard have historically been utilized for packaging food, since they come from trees, they are renewable, and they also biodegrade fairly easily in the right environment. The energy and water consumption associated with the production of paper has led to many instances of deforestation. While recycling paper does mitigate some of these environmental concerns, many paper-based food packages possess a paper outer layer and a plastic inner layer and will be more difficult to recycle once the two layers have dried together; however, advancements in technology for separating these two materials are expected, but not currently in use. Additionally, because of the nature of paper, it is a poor barrier to air and moisture. In turn, this may spoil food more quickly than using plastic; thus, paper is primarily used for dry foods (75).

Packaging selection may be aided by determining the environmental impact of production processes; therefore, a comprehensive assessment of alternative consumption methods may be made. For example, an assessment of production-related carbon and waste footprints associated with various packaging options for a pea-based snack food indicated that packaging alternatives provided no uniform solution for manufacturers (76). While the production of plastic-based food packaging results in a low-profile carbon footprint, the product will ultimately generate a greater quantity of waste than the equivalent alternative produced from either paper or compostable/biodegradable materials (77). Chilled and frozen proteins sourced from alternative protein sources, such as plant proteins or mycoproteins, typically require multiple layers of outer wrapping in addition to the amount of packaging required for the finished product. Using mono-materials across all layers of outer packages has proven to significantly aid recycling programs, making it feasibly possible to recycle the entire product package in one recycling bin, rather than separate bins for each material type (55).

Packaging innovation is another way active packets improve the food supply chain (78). Active packets engage with foods to prolong the shelf life of food products, reducing the amount of food discarded (a serious environmental challenge) while also conserving the resources required to produce that food. Active packets provide another, more indirect, benefit to the environment. However, companies must ensure that active components are safe and that they themselves are also sustainable (79). In recent consumer research, consumers want less plastic packaging, desire recyclable packaging, and claim to be willing to pay a slight premium for sustainable packaging; however, they also want affordable food products (80). Thus, vendors must consider these aspects when developing packaging solutions. One way to reduce the environmental impact of food packaging is to reduce the total quantity of food packaging. Small, incremental changes can yield large collective results (67).

## 6 Consumer acceptance and market dynamics

People's willingness to use alternatives to traditional sources of protein is essential for a sustainable food system to be effective. A good number of consumers want to give alternative proteins a shot; surveys indicate strong interest in plant-based meat products throughout both North America and Europe, with approximately 30%−40% of people having tried these types of meat. Additionally, around 10%−15% of respondents consume them regularly. Consumers also seem to have greater acceptance rates of plant-based milk than other alternatives, with approximately 50% of consumers reporting that they occasionally purchase these products. Product acceptance does vary significantly, as consumers appear to have a much greater desire to sample plant-based hamburgers as compared to insect-based chips or to try out consumption of yogurt made with microbes vs. that which is made with proteins derived from bacteria (14).

What's driving acceptance? Taste! The number one reason people will keep buying a product is if it's tasty. The taste of the product is terrible; people won't buy it again! Therefore, product quality is the most important factor! The second factor is price. Many alternative proteins (i.e., meat substitutes) cost more than traditional meat. For example, in certain countries today, some brands of plant-based milk are less expensive than cow's milk.

On the other hand, plant-based meat costs more than animal-based meat, but the difference is shrinking (81). A third driver of acceptance is health. Many people are interested in alternative proteins for health reasons, such as to lower their saturated fat and cholesterol intake, increase their fiber intake, and avoid the hormones and antibiotics that are used in animal agriculture. One way they can do this is to use simple ingredients and keep ingredient lists short (82).

Some individuals have a profound concern about the environment; these consumers are typically referred to as LOHAS (Lifestyles of Health and Sustainability) the consumer that believes the planet is important and will pay extra money for eco-friendly products. Additionally, they actively seek out sustainability information. The LOHAS market is increasing; however, it still accounts for a minority of the population. The majority of consumers possess some degree of environmental awareness but are not motivated enough to change their consumption patterns (83). There are several barriers to insect consumption. One of the primary reasons for a reluctance to adopt insect consumption is disgust. Consumers have also expressed concerns over their lack of knowledge regarding cooking methods for alternative proteins; they are more accustomed to cooking with animal proteins. For example, while a traditional hamburger may sizzle on the grill, a plant-based burger will not, and it may stick to the cooking surface. Providing simple cooking instructions and recipe ideas can support increased consumption of alternative proteins (84).

There have been massive changes in market dynamics over the past few years. The global market for alternative protein is expected to grow from approximately $15 billion in 2024 to more than $50 billion by 2030. Plant-based meat is the largest segment of this growth; however, other segments are growing at a much higher rate. Cultured meat is still a new and emerging category that has generated significant interest and has high growth potential. In addition, the microbial protein category is also experiencing significant growth. There has been considerable investment in the alternative protein sector, with more than $3 billion of venture capital investment in 2024 alone. This includes investments in research and development (R&D), scaling operations, and marketing efforts (17). There has, however, been some deceleration in growth rates for certain alternative protein products. Also, “plant-based fatigue” has set in with a large percentage of consumers, which has resulted in some consumers feeling “guilty” or pressured by brands to eat plant-based meats. Therefore, the marketing of plant-based meats needs to be designed to be positive rather than preachy (17).

The food service industry is a major channel as restaurants and cafes introduce consumers to new types of food, such as plant-based burgers that someone might not buy at the grocery store but would be willing to try when they are out at a restaurant. If the consumer enjoys the burger at the restaurant, they may purchase one at the grocery store later. Therefore, working with restaurants on these types of meals is important because fast food chains like Burger King and McDonald's are offering plant-based meal options to customers, providing visibility and normalization of plant-based meals (85). Table 3 provides a summary of the different types of consumer segments and their characteristics to aid in targeting marketing.

**Table 3:** Consumer segments for alternative proteins.

Segment	Size (approx.)	Main drivers	Main barriers	Preferred products	References
Enthusiasts	10%−15%	Environment, health, animal welfare	None; already buying	All types, including novel	(14, 83)
Health-focused	20%−25%	Personal health, nutrition	Taste, price	Plant-based milks, tofu	(12, 82)
Price-sensitive	30%−40%	Low cost	High price, unfamiliarity	Cheap options like beans	(18, 81)
Meat-lovers	20%−30%	Taste, tradition	Taste, texture, social norms	Only if similar to meat	(15, 84)
Rejectors	5%−10%	No interest	Disgust, ideology	None	(49)

## 7 Policy, regulation, and economic aspects

Alternative proteins cannot be successful by themselves, so they need government support. The development of policies influences the food system - including what is produced, what is consumed, and the prices. Milk prices are also supported by the production of milk through government subsidies, which means that animal products are cheaper than they should be. The subsidies do not reflect the environmental cost of producing animal products. This is a market failure. With beef, the price does not include the cost of polluting the environment. The price does not include the amount of water required to produce beef. The result is that beef appears to be cheaper than its true cost. Conversely, alternative proteins do not receive these same subsidies; therefore, they are at a competitive disadvantage (86).

One of the ways to overcome this situation is to switch subsidies. Instead of providing money toward feeding animals, the government could provide money to farmers who grow crops for protein, such as peas, lentils, or fava beans. By providing subsidies for alternative protein producers, alternative proteins become more affordable. Any product can be labeled with any name; however, these labels must not be misleading. One example of this would be labeling plant-based milk as “milk”. According to the European Union, plant-based products cannot be referred to as “milk” because “milk” is reserved for cow's milk. Therefore, plant-based beverages are called soya drink, or almond drink, etc. The same rules apply to yogurt and cheese. The dairy industry wants to maintain control of its product definitions; however, plant-based producers believe that when someone refers to they use the term' soymilk,' they understand exactly what it means. Compromises have been made by some countries in allowing manufacturers to create new names for their products (e.g., plant-based milk labeled as “milk,” or beverages labeled as plant-based). Policies must also support farmers transitioning away from livestock (87).

Safety regulations need to exist for alternative protein sources, as well as for new types of food that are being introduced to consumers. For example, in order to receive novel food approval from the EU, it typically takes between 1 and 2 years to secure such approval and costs millions of euros. As a result, small businesses often struggle to obtain novel food approvals. While some people believe this process is too rigorous, others argue that it is needed to ensure the safety of consumers. Risk-based approval would be an option for the approvals. Low-risk products would receive quicker approvals, and high-risk products would be subject to thorough evaluation (4). Policies surrounding trade are also important. Different countries are producing a high amount of different plant-based proteins. For example, Canada produces a significant amount of plant-based protein in the pea form; fava beans are grown extensively in the EU; and China produces a large majority of the world's soy beans. Barriers in the form of tariffs placed on these products would hinder the transition from traditional animal-based sources of protein to plant-based sources of protein. If tariffs were removed from these products, then their price would decrease (20). The EU-specific regulations for novel foods and labeling of plant-based alternatives (88). Challenges include reliance on monocultures (e.g., soy, wheat) that may undermine biodiversity if not managed sustainably.

Economic aspects are costs, the number of jobs, and investment. As production of alternative proteins becomes less expensive, we are nearing price parity between plant-based meat alternatives and traditional meat sources. For example, plant-based sausage is now approximately the same price as regular pork sausage in Germany; however, price parity for plant-based burgers in the US can still be found at 20%−30% more compared to conventional beef. As with all consumer goods, the increased volume of production will result in lower pricing due to economies of scale. The larger the factory, the lower the cost of production. New technologies, such as precision fermentation, are also decreasing in cost. According to some industry analysts, price parity will be achieved within 5 years (18). Employment in the livestock sector is a major area of concern. The livestock industry currently employs a large workforce; however, the growth of alternative proteins may result in substantial job loss for current livestock industry employees. Governments can assist displaced workers with job retraining, as well as support rural communities. The idea of a “just transition” originated in the energy sector and can easily be transferred to the food sector (89). Cultured meat faces particularly high regulatory hurdles (90).

Investment activity in alternative protein sources is on the rise. Venture capital, large corporations through R&D departments, as well as federal government grants/assistance programs, are increasing. The Biden Administration has allocated $10 million of federal funding toward research related to the development of alternative protein sources; the European Union (EU) also has multiple programs aimed at supporting research into alternative protein sources. There has been a significant amount of private investment; one company alone raised $400 million in 2024 for its precision fermentation process. All this investment is leading to the rate of innovation to increase substantially; factories are being built and funded along with consumer market research, which is essential for the growth of the alternative protein sources industry (17). The alternative protein industry is currently experiencing a wave of consolidation, with larger, more established companies acquiring smaller companies that produce alternative protein products. Consolidation when an industry is young is normal. Long-term predictions say that the industry will continue to grow due to increased consumer demand and not simply due to being a trend (91).

## 8 Future perspectives and research directions

The outlook for alternative protein sources in the United States is positive; however, there are still many unknowns in this area. Long-term studies monitoring the eating habits of individuals who consume alternative proteins and tracking cardiovascular outcomes are needed. Bioavailability is impacted by a number of factors, including the food matrix and cooking procedures. Ongoing research will assist in developing the best recipes for the preparation of plant-based meat alternatives.

Technology research is critical to more efficiently producing alternative proteins. While current options, like extrusion, exist, the process requires large amounts of energy. However, advancing technologies such as 3D printing may allow for more realistic meat texture options and custom nutrition. For instance, a 3D-printed steak with additional iron content could be produced specifically for anemic consumers. Another technology being used to create alternative proteins is called precision fermentation.

Additionally, developing better bioreactor designs, such as larger, more efficient tanks, will help reduce the costs of producing alternative proteins. Sustainability research must also continue, and the use of life cycle assessment tools is valuable for determining how sustainable alternative proteins are; however, the current limitations of these tools could affect how sustainable alternative proteins are. For instance, currently, most life cycle assessment studies prepare their assessments based on current energy production and mixes; thus, as energy production transforms to more renewable forms, the estimated contribution of alternative proteins to overall biodiversity will greatly decrease. Furthermore, much of the biodiversity impacts of alternative proteins have not been quantified, but using alternative proteins on a lower land footprint will allow for greater biodiversity, and therefore, quantifying these impacts will be of high importance (92). An additional avenue of research is focused on developing and optimizing circular systems in producing alternative proteins; for example, can we grow microorganisms on CO_2_ emitted from industrial plants? Can we feed insects with food scraps? Assessing these systems can improve the efficiency of production loops within the alternative protein industry. Roy et al. (93) have identified possible transitions between inland aquaculture and semi-intensive fishponds in landlocked Europe (93). Innovative ways to decrease the environmental impact of animal production will be examined (89).

Social science research might go unnoticed, but it is important to help us understand how we can change behavior. What types of messages resonate most effectively? Do scary climate change messages produce effectiveness? Or do positive, health-related messages produce more effectiveness? Research indicates that positive messages resonate more effectively than fear-inducing messages because fear may lead to denial, and hope leads to action. Another area to assess through social science research is cultural adaptation. For example, how can we create alternative proteins that are culturally appropriate? A plant-based hamburger may work well for Americans; however, a plant-based curry may be more appropriate in India.

Additionally, in Japan, it may be more appropriate to create plant-based fish. Research in social science should be used to uncover local ways to create alternative proteins (94). Policy research provides for a better understanding of which policies are most effective at encouraging behavior change, such as carbon taxes, subsidizing alternative proteins, government purchasing through public procurement, or even information campaigns. Each country has different situational contexts (86).

The need for integrated systems is another direction for the future. We must not look at alternative proteins as separate entities; they fit into a much larger food system. We must start to connect crop production, production of animal feed, and production of food for human consumption together through a “One Nutrition” approach. This addresses the entire food production system. For instance, while there may not be good crops suited for human consumption, there are many crops that will be fed to livestock. Livestock can consume insect proteins as well. Thus, all of these components can be tied together. This will allow us to develop better integrated solutions (16). A new and emerging food source is what is referred to as blue food, which is food harvested from the ocean. Examples of blue foods are fish, shellfish, and seaweed. Most, if not all, blue foods are highly sustainable. For instance, the production of mussels and oysters has low impacts on the environment and provides water cleaning services. In addition, seaweed has no requirement for fertilization during growth and has carbon and nitrogen cleaning (uptake) properties as well. The development of seaweed protein as an alternative source of protein is currently underway and promises the potential to be a significant source of protein in the long term (95). However, overfishing of bluefin tuna presents a significant problem. Sustainable aquaculture methods will need to be developed in order to provide sustainable fish sources. Low-trophic (lower in the food chain) fish species will be the best choice for seafood because they do not require wild fish as a source of feed for aquaculture (96).

Cultured meat has been discussed extensively. Cultured meat is produced in laboratories from the cells of animals without having to kill them. It is being studied for its environmental effects as well, although those discussions are varied. Some cultured meat products use fetal bovine serum as their growth medium. The ethical consideration for this type of ingredient is also an important factor in the research currently being conducted. Therefore, researchers are working hard to develop plant-based growth mediums and scale them to the level of commercial production (97, 98). Research that draws upon longitudinal studies is a valuable resource to increase marketing and policy knowledge concerning consumer attitudes toward cultured meat. These studies can assist researchers in developing the most effective methods to improve the marketing and policy development concerning cultured meat products (49). Environmental sustainability of pH-shift technology for recovering proteins from fish side streams (99).

## 9 Conclusion

So far, the article has discussed a lot. It started with how our current food system is damaging the planet. The current food system is damaging because animal products require too many resources and create excessive pollution; it is time to change the food system. Sustainable nutrition systems can improve this situation, as they provide healthy foods while simultaneously creating minimal environmental impacts and providing a solution to sustainable nutrition. The use of alternative proteins will play an important role in these new sustainable nutrition systems; alternative proteins can come from three different sources: plants, microbes, and insects. Each of the alternative protein sources has advantages and disadvantages. Still, alternative protein sources overall will be significantly more sustainable than traditional meat sources. The evidence is clear; using alternative protein sources requires less land, water, and energy; therefore, they produce fewer greenhouse gases than traditional protein sources. There are life cycle assessments that have been conducted that demonstrate that, as an example, plant-based protein is 50%−90% less harmful than beef to the environment. In addition to plant-based protein, microbial and insect-based protein also have lower environmental impacts, similar to or better than plant-based protein. However, there are some considerations to consider as well. Processing alternative proteins can create additional environmental impacts.

Product innovation is rapidly developing with advancements in taste and texture. There is also improvement in nutrient quality, with the addition of vitamins through fortification, and adding protein blends to make an amino acid profile more complete. As well, using less processing energy and utilizing various types of environmentally friendly packaging, including biodegradable, recyclable, and reusable materials, contributes to this effort, based on location. There is increased consumer acceptance, but still a barrier to purchase. The largest barriers tend to be perceived taste/price, as consumers will purchase alternative proteins if they have a similar taste and/or are cheaper.

Additionally, disgust and/or lack of knowledge about alternative proteins are two reasons preventing consumers from buying these products. Education and positive promotion of alternative proteins help, along with the general trend of strong growth in this market; however, many areas have also had slow progress. As the industry matures, strong companies will capitalize and continue to expand.

The food production sector is greatly affected by both the government's economic policies and the market conditions caused by those same economic policies. Methods such as increased diagnostic labeling and food safety standards, improved long-term health assessments of the livestock sector's products, and better ecological assessment will assist in restricting the supply of animal products. The introduction of any new regulations should not create any additional stress on producers of animals. Removing international trade barriers would serve to achieve parity among all producers, regardless of their location. Significant investment is flowing into non-livestock protein sources. Nonetheless, this will limit the amount of resources available for operationalizing the alternative protein sector's products.

Overall, the findings from this review indicate that alternative proteins derived from plants, microbes, and insects have significantly lower environmental impacts (50%−90%) than traditional animal protein sources. Consumers are becoming more willing to purchase alternative proteins, but they face some challenges related to taste, cost, and familiarity. To support a successful market for alternative proteins, governments must create policies such as providing financial incentives (e.g., subsidies) and offering accurate information via clear product labeling. Future research needs to focus onas sessing long-term health effects of consuming alternative proteins, developing production technologies that facilitate large-scale distribution of these products, and culturally appropriate development of alternative protein products.

In conclusion, alternative proteins are not without challenges; rather, alternative proteins will provide access to sustainable methods of food production. Ultimately, alternative proteins will play an integral role in establishing a more sustainable food system. The continued advancement of innovative technologies, supportive government policy directions, increased consumer engagement, and continued investment will be key components in establishing alternative proteins as viable solutions for achieving the new alternative protein economy. Ultimately, there will be a low-impact food production industry, and alternative proteins can be produced in such a way that they are the foundation of that low-impact food production industry.
